# Adenosine A2A Receptor Signaling in the Immunopathogenesis of Experimental Autoimmune Encephalomyelitis

**DOI:** 10.3389/fimmu.2018.00402

**Published:** 2018-03-06

**Authors:** Skanda Rajasundaram

**Affiliations:** ^1^Medical Sciences Division, Oxford University, Oxford, United Kingdom

**Keywords:** adenosine, adenosine 2A receptor, experimental autoimmune encephalomyelitis, multiple sclerosis, neuroinflammation, microglia

## Abstract

Our increasing appreciation of adenosine as an endogenous signaling molecule that terminates inflammation has generated excitement regarding the potential to target adenosine receptors (ARs) in the treatment of multiple sclerosis (MS), a disease of chronic neuroinflammation. Of the four G protein-coupled ARs, A2ARs are the principal mediator of adenosine’s anti-inflammatory effects and accordingly, there is a growing body of evidence surrounding the role of A2ARs in experimental autoimmune encephalomyelitis (EAE), the dominant animal model of MS. Such evidence points to a complex, often paradoxical role for A2ARs in the immunopathogenesis of EAE, where they have the ability to both exacerbate and alleviate disease severity. This review seeks to interpret these paradoxical findings and evaluate the therapeutic promise of A2ARs. In essence, the complexities of A2AR signaling arise from two properties. Firstly, A2AR signaling downregulates the inflammatory potential of TH lymphocytes whilst simultaneously facilitating the recruitment of these cells into the CNS. Secondly, A2AR expression by myeloid cells – infiltrating macrophages and CNS-resident microglia – has the capacity to promote both tissue injury and repair in chronic neuroinflammation. Consequently, the therapeutic potential of targeting A2ARs is greatly undermined by the risk of collateral tissue damage in the periphery and/or CNS.

## Introduction

Multiple sclerosis (MS) is the most common chronic neuroinflammatory disease in the Western World, affecting ~2.5 million people worldwide, typically in the third and fourth decades of life (1). The equipoise between genetic and environmental factors is undoubtedly central to the etiology of MS yet despite years of research, the precise cause of MS remains elusive. Inflammation, demyelination, reactive gliosis, and neuroaxonal degeneration characterize CNS lesions observed in MS patients, and the heterogeneous spatiotemporal dissemination of these lesions is reflected by the heterogeneous clinical presentation of MS. This typically includes some combination of somatosensory and visual defects, impairments in pyramidal-motor control, fatigue, pain, and cognitive deficits.

The immunopathogenesis of MS is characterized as a T cell-mediated autoimmune response against myelin self-antigen, which provokes the migration of immune cells across the blood–brain barrier and blood–CSF barrier (2). Within the CNS, macrophages and T cells (both CD4^+^ and CD8^+^) dominate the inflammatory infiltrate. EAE, the principal animal model of MS, has been fundamental in investigating the immunopathogenic mechanisms underlying MS. This is because it can recapitulate the cardinal pathological features of MS observed in patients, namely inflammation, demyelination, axonal loss and gliosis (3). Experimentally, it is induced by immunizing animals with myelin derived proteins—typically myelin oligodendrocyte glycoprotein (MOG) in mice—which results in the generation of primed CD4^+^ T helper 1 (T_H_1) and T_H_17 cells, which in turn drive EAE pathogenesis. EAE is able to accurately recapitulate the early, *inflammatory* phase of MS, during which a degree of remyelination is possible. However, in the second phase of MS, axonal degeneration commences and remyelination becomes increasingly difficult. This *neurodegenerative* phase is less accurately recapitulated by EAE, which is, after all, immunological in nature. Accordingly, a different set of mechanisms must be considered to explain the distinct, neurodegenerative component of MS.

A handful of immunomodulatory agents have had success in managing relapsing–remitting MS, the most common clinical form of MS. First generation therapies such as IFN-β and glatiramer acetate reduce both the frequency and severity of relapse and have good safety records, but they do not substantially halt disease progression (4). Among the newly developed monoclonal antibody (mAb) therapies, the most notable is alemtuzumab, which is significantly more efficacious in reducing relapse rates than first generation therapies and unprecedentedly, is able to improve long-term disability outcomes; however, concerns regarding the safety profile of alemtuzumab have been raised (5). Furthermore, only one drug—ocrelizumab—has been approved specifically for primary progressive MS, and no treatments have been approved specifically for secondary-progressive MS. Thus, given the magnitude of the disease burden, MS remains a major clinical challenge with scope for novel therapeutic targets and EAE remains instrumental in addressing this challenge.

## Purinergic Signaling in Inflammation

Cellular stress or apoptosis induces the release of ATP into the extracellular space, promoting rapid inflammation by activating ATP receptors of which there are two subtypes, inotropic P2X receptors and metabotropic P2Y receptors (6). Both P2XRs and P2YRs amplify T cell receptor as well as innate immune signaling. Indeed, the potent ability of ATP to promote inflammasome activation in macrophages and dendritic cells renders it an important “damage-associated molecular pattern” in the acute inflammatory response to cellular damage and destruction (7–9).

**Figure 1:**
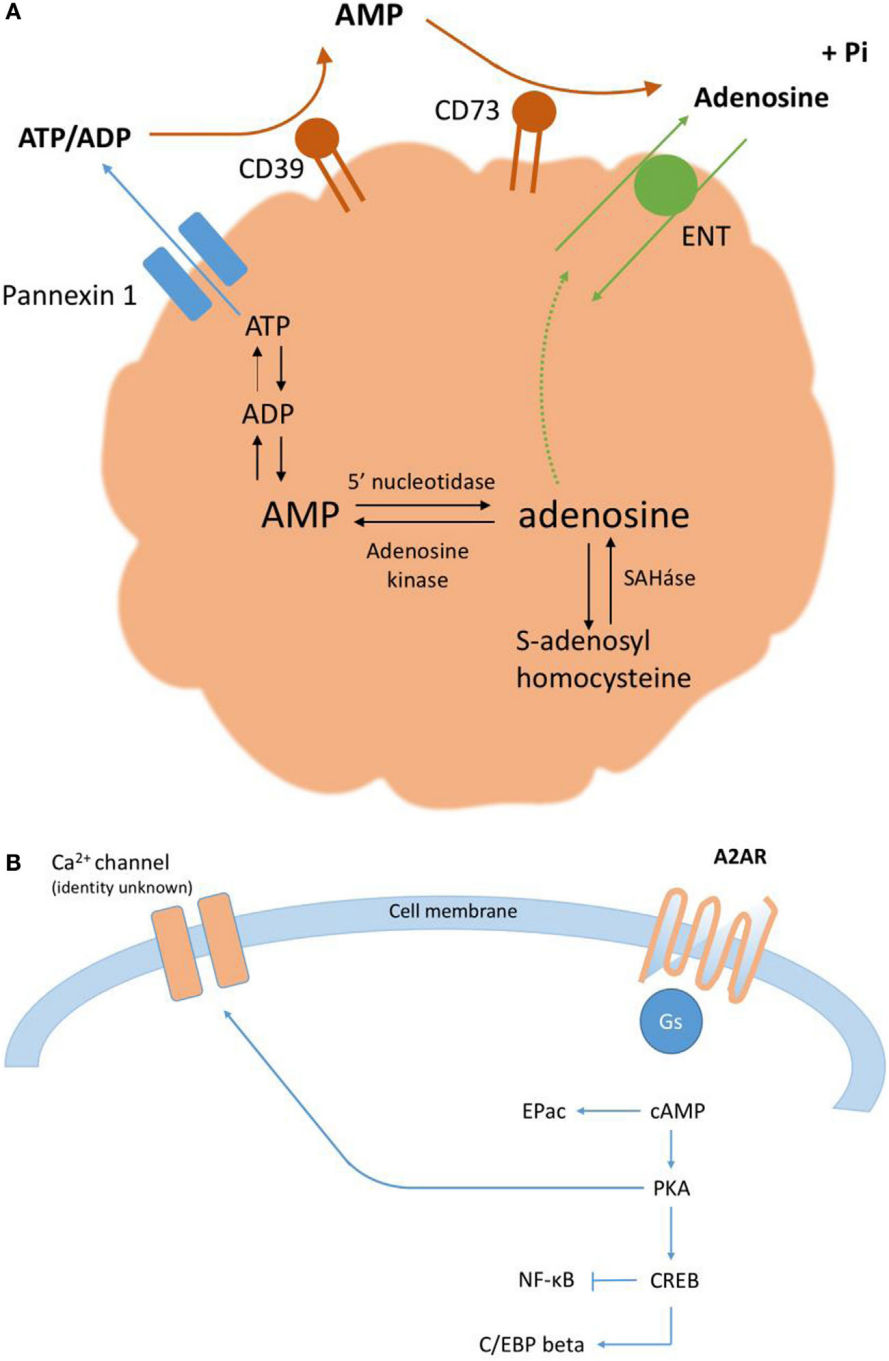
**(A)** Extracellular adenosine accumulates *via* the breakdown of ATP, both intracellularly and extracellularly. **(B)** A2ARs signal predominantly *via* the adenylate cyclase-cAMP-protein kinase A (PKA) canonical pathway (10). PKA phosphorylates the transcription factor CREB on serine residue 133; activated CREB can affect gene expression directly, *via* specific promoters, or indirectly, *via* an important cofactor, CBP. cAMP can also signal directly *via* the exchange factor Epac.

The accumulation of extracellular ATP described above characterizes the acute phase of purinergic signaling, which lasts minutes to hours (6). In the subacute phase of purinergic signaling, lasting days to weeks, the extracellular ratio of ATP/adenosine declines. Correspondingly, there is a reduction in ATP signaling concomitant to an increase in the activation of P1 ARs, which serves to restrict the degree and duration of inflammation. Ordinarily, adenosine that accumulates in the extracellular environment is rapidly taken up *via* nucleoside transporters into neighboring cells, where adenosine is metabolized either by adenosine kinase to form AMP or by adenosine deaminase to form inosine; however, under inflammatory conditions, adenosine removal cannot keep pace with its generation. This increase in extracellular adenosine (Figure 1A), from basal nanomolar concentrations to ~10–50 µM, has potent and well-documented anti-inflammatory effects *via* one or more of four G protein-coupled ARs, denoted A1, A2A (Figure 1B), A2B, and A3. Finally, in the chronic phase of purinergic signaling, the low extracellular ratio of ATP/adenosine is associated with wound healing and can, on occasion, lead to pathological tissue remodeling.

From this temporal analysis, established recently by Cekic and Linden (6), it follows that that the accumulation of extracellular adenosine and activation of P1 ARs increase over time, bearing particular relevance to chronic neuroinflammatory conditions such as in EAE.

A2ARs are widely expressed in the CNS and among the key peripheral immune cells implicated in EAE (Table 1); furthermore, evidence from EAE studies and a range of other inflammatory conditions suggest that A2ARs are the prime mediator of adenosine’s anti-inflammatory effects (11–13). Correspondingly, interest in the role of A2AR signaling in the immunopathogenesis of EAE has blossomed and it has been suggested that A2ARs may offer a novel therapeutic target for MS.

**Table 1:** The expression and function of A2ARs in the CNS and peripheral immune system.

Region of A2AR expression	Functional effects of increased extracellular adenosine/ATP
**Central nervous system**	
– Striatum	Postsynaptic reciprocal inhibitory interactions with D2 receptor signaling in striatopallidal medium spiny neurons (MSNs) involved in locomotor control (14)
	Presynaptic facilitation of glutamate release from cortico-striatal glutamatergic terminals in contact with striatonigral MSNs involved in locomotor control (14)
– Prefrontal cortex	Modulates acetylcholine release and inhibits cortical and behavioral arousal (15)
– Hippocampus	At the cellular level, facilitates excitatory glutamatergic Schaffer collateral synapses to CA1 pyramidal cells (16)
	Behaviorally, optogenetic stimulation of A2AR signaling pathways induces an impairment of spatial memory (17)

**Peripheral immune system**	
– CD4^+^ [T helper 1 (T_H_1) cells]	Anti-inflammatory—inhibits production of a range of cytokines inc. IL-2, TNF-α, and IFN-γ but has little effect on IL-4 and IL-5 production (18, 19)
– CD4^+^ (T_H_17 cells)	Anti-inflammatory—little effect on cytokine production but inhibits development of T_H_17 cells (20)
– Invariant natural killer cells	Anti-inflammatory—inhibits IFN-γ production (20)
– CD8^+^	Anti-inflammatory—mild impairment of proliferation but significant inhibition of IFN-γ and associated cytotoxicity (21)
– T_reg_	Anti-inflammatory—encourages T_reg_ development in naïve T cells. Furthermore, expression of CD39 and CD73 on T_regs_ facilitates increase in adenosine availability (20)
– Macrophages and dendritic cells	Anti-inflammatory—reduces capacity to induce T_H_1 polarization in naïve CD4^+^ T cells, reduces production of pro-inflammatory TNF-α and IL-12, and enhances release of anti-inflammatory IL-10 (20)

**Central nervous system immune system**	
– Microglia	Enables a response to CNS inflammation by triggering process retraction and amoeboid morphology (22)
	Anti-inflammatory—inhibits microglial activation, which is implicated in release of both pro-inflammatory cytokines and reactive oxygen species (23)
	Pro-inflammatory—may facilitate production of inflammatory mediators such as nitric oxide and inhibit remyelination (24)
– Choroid Plexus Epithelium	Pro-inflammatory—may facilitate the transmigration of lymphocytes into the CNS (23)

Of course, an evaluation of the therapeutic potential of A2ARs requires both an overview of A2A receptor regulation and an appreciation of the complex role of A2AR signaling in the progression of EAE. Underlying these complex effects are, firstly, the paradoxical effects of A2AR signaling in the recruitment of lymphocytes to the CNS and, secondly, the paradoxical effects of A2AR signaling in both infiltrating macrophages and CNS-resident microglia, during chronic neuroinflammation. Importantly, this interpretation must be evaluated against the limitations of EAE as an animal model of MS, with an emphasis on those limitations that apply to A2AR signaling in particular.

## A2A Receptor Regulation

The molecular basis of A2A receptor regulation was investigated in pioneering studies into A2AR gene structures, which were shown to be highly conserved across mice, rats and humans (10, 25). The A2AR gene is composed of multiple exons that encode alternative transcripts, which are initiated from at least four independent promoters. Of the transcripts identified to date, they share identical coding regions and a common 3′ untranslated region (UTR) but distinct 5′ UTRs; thus, the function of these distinct 5′ UTRs is of particular interest in elucidating A2AR regulatory mechanisms. 5′ UTRs corresponding to the P2 and P3 A2AR promoters appear to suppress A2AR expression at the translational level while the regulatory function of 5′ UTRs that correspond to the P1A and P1B A2AR promoters is unclear. Moreover, transgenic studies in rats suggest that P1A, P2, and P3 promoters are responsible for A2AR expression in the CNS (26), which raises the possibility that the P1B promoter might regulate peripheral A2AR expression. Looking forward, it will be important to identify the DNA elements underlying the intense expression of A2ARs in the striatum, which have not been recapitulated by the transgenic approach employed in these experiments.

In states of both inflammation and chronic neurodegenerative disease, changes in A2AR expression are well documented. In both murine and human macrophages, lipopolysaccharide induces an increase in A2AR mRNA expression in an NF-κB-dependent manner (27). By contrast, in Huntington’s disease, the mutant Huntingtin gene exerts transcriptional suppression of striatal A2ARs *via* CREB inhibition (28). However, changes in A2AR expression in MS, which is characterized by both chronic neuroinflammation and neurodegeneration, and exhibits subtler genetic mechanisms, remain poorly characterized.

Thus, further characterization of the A2AR gene is necessary if we are to understand the molecular basis of how A2A receptors are physiologically regulated and indeed how they can be pharmacologically manipulated under pathophysiological conditions for therapeutic purposes.

## A2AR Signaling has Paradoxical Effects on Lymphocyte Recruitment to the CNS during EAE

The most direct evidence of a role for A2ARs in the immunopathogenesis of MS comes from EAE animal studies, in which it has been shown repeatedly that knocking out A2ARs exacerbates the severity of EAE, as evidenced by greater motor paralysis, more infiltrating CD4^+^ T lymphocytes in the CNS and more demyelination in A2AR KO (A2AR^−/−^) mice in comparison with WT mice (23, 29). A possible confound in A2AR^−/−^ mice induced with EAE is the loss of neuronal A2AR expression in the dorsal striatum and so the motor paralysis observed in EAE A2AR^−/−^ mice may in part be attributable to impaired striatal motor control, in addition to the expected loss of A2AR signaling in the immune system. Importantly, therefore, the results of genetic knockout studies have been validated by pharmacological studies in lymphocytes isolated from MS patients (18). Stimulating A2ARs with the A2AR agonist CGS21680 significantly inhibits lymphocyte proliferation, VLA-4 expression and the release of a range of pro-inflammatory cytokines, including TNF-α, IFN-γ, IL-6, IL-1-β, and IL-17, all of which have been shown to contribute to MS progression (19, 30).

However, in direct contrast to these observations, it has been repeatedly shown that pharmacologically antagonizing A2ARs with SCH58261 confers protection against the induction of EAE in WT mice (23, 31). This contradiction was investigated in a series of elegant adoptive transfer experiments using the radiation bone marrow chimera model system (23). Adoptively transferring A2AR^−/−^ CD4^+^ T lymphocytes into A2AR^+/+^ tcr-deficient mice induced an EAE pathology more severe than when WT CD4^+^ T lymphocytes were adoptively transferred into A2AR^+/+^ tcr-deficient mice and even, crucially, more severe than when A2AR^−/−^ CD4^+^ T lymphocytes were adoptively transferred into the A2AR^−/−^ phenotype. Furthermore, the transfer of A2AR^+/+^ lymphocytes into A2AR^−/−^ mice did not induce EAE and importantly, neither FoxP3^+^ immunostaining nor T_effector_ suppression assays suggested any confounding alterations in T_reg_ frequency and/or functionality in A2AR^−/−^ mice. These findings suggest that A2AR expression in T_H_ lymphocytes is essential for limiting the severity of the inflammatory response in EAE, while A2AR expression on radiation-resistant, non-hematopoietic cells promotes severe EAE. Thus, while knocking out A2ARs increases susceptibility to developing severe EAE due to the increased pro-inflammatory nature of A2AR^−/−^ lymphocytes, it appears that SCH58261’s blockade of A2ARs is protective *via* its action on non-hematopoietic cells.

The non-hematopoietic cells of interest are the choroid plexus epithelium (CPE), in which fluorescence *in situ* hybridization (FISH) studies reveal a high degree of A2AR and CD73 mRNA expression. Furthermore, this structure is an established CNS entry point for immune cells in MS (32–34). However, FISH does not functionally demonstrate the capacity of CPE A2AR signaling to mediate lymphocyte transmigration in EAE.

Another study compared the effects of introducing an A2AR agonist, CGS21680, at different time points following MOG immunization in an attempt to investigate how the role of A2AR signaling changes throughout the course of EAE (35). Introducing CGS21680 on the day of MOG immunization reduced the severity of EAE and consistent with previous findings, adoptive transfer experiments demonstrated the mechanism of such protection to be the downregulated inflammatory potential of A2AR-expressing lymphocytes. However, introducing this CGS21680 12 days postimmunization (i.e., at the peak of the disease) exacerbated the severity of EAE in comparison with vehicle-treated mice. One possible limitation of this study is that these effects were not shown to be reversible with a selective A2AR antagonist. Indeed, given that A2AR–A2BR heterodimerization has been documented (36), and knocking out A2BRs also exacerbates the severity of EAE (37), it is possible that CGS21680 exerted confounding non-specific effects on A2BRs.

While Ingwersen et al. do not offer an interpretation of this paradox, it is plausible that the opposing effects of A2ARs signaling in T_H_ lymphocytes vs non-hematopoietic cells may account for these remarkable observations, especially since in the disease course of EAE, the peripheral activation of T_H_ cells occurs primarily in the first week post-immunization and by day 12, immune cells have begun to infiltrate the CNS in increasing numbers (5). To substantiate this correlation between the time-dependent effects of CGS21680 and the A2AR-sensitive progression of EAE, it would be necessary to investigate whether the stage of EAE at which an A2AR agonist is introduced affects the infiltration of adoptively transferred lymphocytes into the CSF.

## A2AR Signaling in T_reg_ Lymphocytes in EAE

The significance of A2AR signaling in lymphocytes in particular is further supported by its ability to shape the immune response *via* T_reg_ control. This is because FoxP3^+^ T_reg_ cells are unique among T cells in their surface expression of both CD39 and CD73 ectoenzymes and thus, in their ability to generate pericellular adenosine from extracellular ATP and ADP (38). This, in addition to the marked expression of A2ARs on T effector cells, places A2AR activation at the center of T_reg_-mediated immunosuppression.

Indeed, the augmentation of T_reg_-mediated immunosuppression can alleviate variants of EAE (39). For example, in both C57Bl/6 and SJL recipient mouse strains, which model chronic and relapsing–remitting forms of MS, respectively, it has been shown that passively transferring peripheral CD4^+^ CD25^+^ T cells from mice with EAE suppresses the development of chronic EAE in recipient mice (40). Similarly, passively transferring a small number of CNS-derived T_reg_ cells isolated from mice in the recovery phase of EAE considerably alleviated MOG-induced EAE in recipients (41). Interestingly, passively transferring an identical number of CD4^+^ CD25^+^ T cells from lymph nodes did not alleviate EAE in recipients. The greater capacity of CNS-derived T_reg_ cells to downgrade inflammation in comparison with peripheral T_reg_ cells highlights the importance of antigen specificity in T_reg_-mediated immunosuppression in classical MOG-induced EAE. Furthermore, in the Tg MBP/Rag^−/−^ EAE mouse model—in which transgenic mice expressing a TCR against myelin basic protein are crossed to mice of a recombination-activation gene 1—deficient background—T_reg_ cells are central in the resistance to EAE development in Tg MBP/Rag^+/+^ mice (42). Further to this, it was shown that adoptively transferring CD4^+^ CD25^+^ T cells to Tg MBP/Rag^−/−^ mice engenders resistance to spontaneous EAE development. In addition to these findings from EAE studies, an increasing amount of evidence supports a role for T_reg_ cells in MS in which CD4^+^ CD25^high^ T_reg_ cells may be functionally impaired in their maturation and emigration from the thymus (43–46).

In summary, A2AR signaling is of central importance in T_reg_-mediated immunosuppression and T_reg_ cells have been demonstrated to mitigate against the development and progression of a range of EAE models. A direct investigation into the role of T_reg_ A2AR signaling in EAE, perhaps involving the conditional genetic deletion of these receptors in CD4^+^ CD25^+^ T cells, is an obvious next step in understanding lymphocytic A2AR signaling in the context of EAE.

## A2AR Signaling Potently Regulates Monocyte/Macrophage-Derived TNF-α, which has Contrasting Effects in EAE and MS

A2ARs are highly expressed on infiltrating macrophages, which predominate in lesions in both EAE and MS, and the numbers of which correlate to tissue damage (6). In murine monocytes, knocking out A2ARs produces a significant upregulation of TNF-α production (47) while stimulating A2ARs with CGS21680 produces a significant downregulation of TNF-α production (48). These findings are validated by clinical observations of elevated CSF levels of TNF-α (49) and reduced plasma levels of cAMP in MS patients in comparison with control subjects (50). Furthermore, administering antibodies that neutralize TNF-α has been shown to abrogate EAE development (51) and the overexpression of TNF-α in transgenic mice results in lesions of demyelination mirroring those observed in MS patients (52). This suggests a role for TNF-α in potentiating demyelination. Unexpectedly, however, the TNF-α receptor blocker Lenercept was found to dose dependently increase the frequency of relapse in MS patients in phase II clinical trials (53), suggesting that TNF-α plays a more complex role in MS. Further investigations in oligodendroglia found that TNF receptor I mediates nerve demyelination whereas TNF receptor II is essential to nerve remyelination. Indeed, the expression of TNF receptor II alone was sufficient to restore oligodendrocyte regeneration in TNF-α^−/−^ mice (54). Thus, it is difficult to envisage the A2AR-mediated modulation of TNF-α release as a promising therapeutic avenue given that this cytokine can promote both the progression and regression of MS depending on the TNF receptor subtype it activates.

A2ARs also upregulate the release of IL-10, an anti-inflammatory cytokine that acts directly on CD4^+^ T cells, inhibiting proliferation as well as the release of TNF-α, IL-2, IFN-γ, IL-4, and IL-5. It has been shown that IL-10 levels are reduced in MS patients and restoring them back to physiological levels may be one of the elusive therapeutic mechanisms of IFN-β-1b (55). Thus, inducing IL-10 release *via* A2AR agonism could compliment IFN-β-1b treatment. However, in light of the contrasting effects of TNF-α in MS, directly administrating IL-10 may be a more promising therapeutic avenue than A2AR modulation.

Interestingly, the control of monocyte/macrophage-derived TNF-α by A2AR signaling has elucidated the importance of oxygen availability in the recruitment of the adenosine signaling system. It has been shown repeatedly that A2ARs are instrumental in the downregulation of TNF-α in murine macrophages in response to hypoxia (56, 57), a switch that involves the induction of HIF-1α by TLR4 activation and post-transcriptional stabilization of HIF-1α by A2AR signaling. The link between HIF-1α and A2ARs and more generally, the increased adenosine release by cells in hypoxic environments, suggests oxygen availability could be a fundamental trigger in recruiting adenosine signaling. Indeed, in light of recent findings, this may have relevance to the immunopathogenesis of EAE. Using novel fiber-optic PO_2_ sensors, oxygenation in cortical and cerebellar gray matter was quantified in awake, unrestrained mice with MOG-induced EAE (58). Both cortical gray matter and cerebellar gray matter were hypoxic, and cortical gray matter hypoxia correlated with behavioral deficits. Of course, considering the contrasting effects of hypoxia-related inflammatory mediators such as monocyte/macrophage-derived TNF-α in white matter lesions, it is unclear whether A2AR signaling sustains or alleviates gray matter inflammation in EAE. Thus, further characterization of A2AR signaling in the context of hypoxia-related gray matter inflammation is warranted.

## Microglial A2AR Signaling has Contrasting Effects in EAE

Similar to infiltrating macrophages, microglia also have the ability to promote both tissue injury and repair (59, 60), and A2ARs appear capable of facilitating both of these contrasting effects.

A number of studies have linked microglial activation in EAE to demyelination, the release of pro-inflammatory cytokines and the production of reactive oxygen species. Consistent with these findings and the protective, anti-inflammatory effects of A2ARs observed elsewhere, it was recently shown that the more severe EAE phenotype in A2AR^−/−^ mice exhibited more Iba1^+^ cells [Iba1 is a specific marker of microglial activation (61)] than WT mice in post-mortem sections (23). However, this observation does not distinguish between the enhanced microglial activation resulting from the increase in TNF-α release by infiltrating macrophages and lymphocytes that now lack A2ARs, and the enhanced microglial activation resulting from the absence of microglial A2AR signaling. Moreover, this finding is potentially confounded by developmental changes in A1R expression in A2AR^−/−^ mice, given that A1Rs are expressed in microglia and especially since A1R KO studies have implicated these receptors in EAE progression (62).

By contrast, evidence from cultured microglial cells indicates microglial A2AR signaling has the capacity to exacerbate EAE. CGS21680 concentration dependently potentiates LPS-induced nitric oxide (NO) and NO synthase-II expression, both of which characterize the microglial inflammatory response (24) and indeed, A2AR blockade curtails LPS-induced microglia-mediated neuroinflammation (63). Furthermore, the exposure of macrophages and microglia to myelin debris *in vitro* leads to an upregulation of A2AR expression in these cells and subsequent CGS21680 treatment inhibits the cellular uptake of myelin debris (35), a well-documented prerequisite for remyelination (64–66). This is corroborated by other studies showing that A2AR stimulation reduces the uptake of fluorescein-labeled *E. coli* bioparticles by LPS-treated microglia (22). Thus, microglial A2AR signaling may be capable of both reducing and exacerbating the severity of EAE due to the complex role of microglial cells in CNS inflammation.

For a long time, investigating the effects of microglia on the progression of EAE has been limited by our ability to distinguish microglia from other myeloid cells. Recently, however, a specific marker of microglia, transmembrane protein 119 (Tmem119), has been identified in both mice and humans, using *in situ* hybridization and qPCR analyses (67). Crucially, FACS studies have shown that Tmem119 distinguishes microglia from infiltrating macrophages in various models of CNS inflammation. Thus, using Tmem119 promotor-driven Cre-recombinase mouse, it may soon be possible to compare the progression of EAE in the presence and absence of microglial A2AR signaling.

## EAE is a Useful but Reductive Model of MS

Our understanding of the role of A2AR signaling in the immunopathogenesis of MS is derived almost entirely from MOG-induced EAE studies, which have a number of limitations (5) (Table 2).

**Table 2:** The limitations of experimental autoimmune encephalomyelitis (EAE).

Feature of EAE	Limitation(s) in recapitulating multiple sclerosis (MS)	Possible solution
Low immunogenic potential of myelin oligodendrocyte glycoprotein (MOG) necessitates administration of strong adjuvants including complete Freud’s adjuvant and pertussis toxin	Intense innate immune response to these stimuli in EAE may not reflect pattern recognition in MS	Spontaneous EAE models have been recently established in both the C57BL/6 background and the SJL/J background

MOG-dependent EAE is typically induced in C57BL/6 mice, in which EAE exhibits a chronic, monophasic disease course	Does not reflect the typically relapsing–remitting nature of MS observed clinically	More frequent use of SJL/J strain, which can develop relapsing–remitting EAE

EAE is driven primarily by CD4^+^ T cells	Underplays roles of CD8^+^ T cells, which outnumber CD4^+^ T cells in cortical demyelination lesions in MS, and antigen-experienced B cells, which have been shown to undergo affinity maturation in cervical lymph nodes before migrating to CNS	Corroborate findings with studies using models not driven primarily by CD4^+^ T cells, e.g., cuprizone feeding and Theiler’s virus infection

Most importantly, MS has increasingly been recognized to have a progressive neurodegenerative component that is independent from its autoimmune component and comparable to aspects of Parkinson’s disease (4). Accordingly, the greatest limitation of EAE may be its bias toward the immunological component of MS pathophysiology, as illustrated by microarray gene expression profiles, which reveal more changes in immunologically relevant genes in EAE than in MS (68). Indeed, MS has historically been considered a T_H_ cell-mediated pathogenesis *because* EAE is driven by CD4^+^ T cells and accurately recapitulates several features of MS. Consequently, the importance of A2ARs in the pathogenesis of MS may be inflated by modeling MS with EAE, where A2AR signaling exacerbates disease *via* its effects on immune cells and immune cell transmigration. However, given the importance of oligodendroglia in regulating remyelination, the recent finding that stimulating surface A1Rs and A2ARs dose dependently causes oligodendroglial death (69) may also implicate A2AR in the neurodegenerative elements of MS.

Indeed, it is possible that neuronal A2ARs might mediate the transition of MS from a disease of neuroinflammation to one of irreversible neurodegeneration. In this regard, A2ARs appear to control the impact of neuroinflammatory mediators on neuronal viability (70, 71) and in different animal models of Alzheimer’s disease, A2AR blockade provides neuroprotection at least in part by preventing damage to axon terminals (72, 73). These findings are supported by small-scale clinical studies in which dynamic positron emission tomography imaging of secondary-progressive MS patients, using a radioligand to A2ARs, demonstrate an upregulation of A2ARs in normal-appearing white matter (74). Future studies should investigate the role of A2AR signaling in MS-related neurodegeneration, perhaps using alternative models of MS such as cuprizone feeding (75), which better recapitulates cortical demyelination.

## Therapeutic Potential of A2A Receptors in MS

The potential for A2ARs to serve as therapeutic targets in the treatment of MS is frequently alluded to in the literature. For example, administering the A2AR antagonist SCH58261 protected MOG-immunized A2AR^+/+^ tcr-deficient mice from developing EAE both upon the adoptive transfer of WT and upon the transfer of A2AR^−/−^ CD4^+^ T cells, demonstrating that A2AR antagonist-mediated blockade is protective even in the presence of more pro-inflammatory A2AR^−/−^ CD4^+^ T cells (23). As discussed earlier, this likely demonstrates the inhibitory effects of A2AR antagonists on non-hematopoietic cells, in which A2AR signaling facilitates the lymphocyte migration into the CNS. However, given that A2AR signaling also downregulates the inflammatory potential of T_H_ lymphocytes, the potential benefits of inhibiting CNS infiltration cannot be elicited without concomitantly increasing the pro-inflammatory nature of T_H_ lymphocytes, which risks causing toxic side effects given that adenosine signaling is so widespread in the body and is involved in a range of physiological functions. Indeed, the fine line between the protective and harmful effects of a given A2AR-specific agent has been demonstrated *in vivo*, whereby administering an A2AR agonist on the day of MOG immunization confers protection yet administering the A2AR agonist during the peak of the disease exacerbates EAE (35). Moreover, EAE and MS aside, AR-specific agents have historically struggled to reach the clinic because developing viable AR-specific agents that exhibit tissue selectivity and an appropriate *in vivo* biodistribution is fundamentally challenged by the ubiquity of adenosine signaling in the body (76, 77). In MS, however, the challenge of selectively targeting A2ARs is complicated by the paradoxical effects of these receptors. Thus, targeting A2ARs directly may not have the therapeutic promise that many have hoped for.

Nevertheless, establishing the important role of A2AR-mediated lymphocyte recruitment to the CNS may yield other viable therapeutic opportunities. For example, RT-PCR analysis shows that the expression of CX3CL1 (a chemokine/adhesion molecule) is upregulated in the CPE during EAE (78). CGS21680 also dose dependently increases CXCL1 expression in CPE cell lines. Although it must first be experimentally demonstrated that A2AR-dependent CXCL1 activity increases the infiltration of lymphocytes into the CSF, as indicated by preliminary findings, targeting CXCL1 directly with monoclonal antibodies, rather than *via* A2ARs, may be a viable therapeutic avenue that overcomes the opposing effects of A2AR expression on different cell types. Furthermore, CXCL1 mAb therapies could offer a more precise therapeutic alternative to alemtuzumab, which, while unprecedentedly efficacious, also causes severe immunosuppression that can lead to acquired autoimmune deficiency (79).

As our understanding of the role of T_reg_ A2AR signaling in EAE deepens, this may offer a new avenue for an immunotherapy that is capable of slowing the progression of MS; indeed, there is indirect evidence in support of this. Recently, it was shown that tolerance-inducing gene immunotherapy was able to prevent the onset and progression of MOG-induced EAE (80). By using a liver-targeting gene transfer vector to ectopically express MOG in hepatocytes, functional FoxP3^+^ T_reg_ cells were induced to expand *in vivo*, and in turn engender tolerogenicity to MOG. This elegant experiment highlights the potential of T_reg_–related therapies and suggests that the cell types in which A2AR signaling is particularly potent and indeed, demonstrably abrogates CNS inflammation, might offer a therapeutic target more viable than targeting A2AR receptors directly.

Caffeine, a non-selective antagonist of ARs, has been shown to provide protection in MOG-induced EAE. Here, however, it is likely that A2ARs play a minor role in comparison with A1Rs given that caffeine administration is able to reverse EAE pathology in A2AR KO mice and chronic caffeine treatment upregulates A1 receptors but not A2ARs (81). In line with A1R upregulation, an upregulation of TGF-β and a downregulation of IFN-γ mRNA has been observed in Wistar rats induced with EAE (82), consistent with an A1R-mediated shift in T_H_1 to T_H_2 function. Furthermore, as a readily consumed psychoactive drug, caffeine avoids some of the inherent challenges that AR-specific agents face in reaching the clinic. Epidemiologically, however, the evidence in humans is mixed, with one study showing that caffeine consumption is not significantly associated with the risk of developing MS (83) whereas a more recent analysis suggests that a high caffeine intake is associated with risk reduction (84). In any case, with respect to possibility of caffeine treatment in humans, A1Rs may be of greater therapeutic importance than A2ARs.

In light of our increasing appreciation of the neurodegenerative component of MS, therapeutic prospects might be informed by insights into other neurodegenerative diseases. It has been shown that excessive A2AR activity is implicated in the development of memory deficits in animal models of Alzheimer’s disease (85–87). Consistent with this, both A2AR antagonists and regular consumption of moderate doses of caffeine prevent memory dysfunction arising in a range of conditions, including Parkinson’s disease (88), Huntington’s disease (89), chronic stress (90), aging (91), early-onset convulsions (92), and diabetic encephalopathy (93). Recently, A2AR inactivation has been found to alleviate early-onset cognitive dysfunction following traumatic brain injury and conditional genetic inactivation of astrocytic A2ARs enhanced long-term memory in the hAPP mouse model of Alzheimer’s (94). Thus, even if blocking A2ARs cannot provide a simple solution to managing the progression of MS, it may yet offer some meaningful symptomatic relief and in turn improve patients’ quality of life (95).

## Concluding Remarks

In contrast to the anti-inflammatory effects of A2AR signaling in the periphery, which serve to restore tissue homeostasis in response to metabolic stress and cell damage, A2ARs are capable of both facilitating and inhibiting the progression of CNS inflammation. Consequently, A2AR signaling exerts paradoxical effects in the immunopathogenesis of EAE, which in turn undermines the therapeutic potential of these receptors in MS. Even so, unraveling the potent albeit complex effects of A2ARs in EAE, may yet be of instrumental value in revealing novel therapeutic opportunities, which can selectively harness the protective mechanisms induced by A2ARs without targeting these receptors directly.

## Author Contributions

The author confirms being the sole contributor of this work and approved it for publication.

## Conflict of Interest Statement

The author declares that the research was conducted in the absence of any commercial or financial relationships that could be construed as a potential conflict of interest.
